# Young children with Down syndrome show normal development of circadian rhythms, but poor sleep efficiency: a cross-sectional study across the first 60 months of life

**DOI:** 10.1016/j.sleep.2016.12.026

**Published:** 2017-05

**Authors:** Fabian Fernandez, Casandra C. Nyhuis, Payal Anand, Bianca I. Demara, Norman F. Ruby, Goffredina Spanò, Caron Clark, Jamie O. Edgin

**Affiliations:** a Departments of Psychology and Neurology, BIO5 Institute, University of Arizona, Tucson, USA; b Evelyn F. McKnight Brain Institute, University of Arizona, Tucson, USA; c Department of Psychology, University of Arizona, Tucson, USA; d Sonoran University Center for Excellence in Developmental Disabilities, University of Arizona, Tucson, USA; e Biology Department, Stanford University, Stanford, USA; f Department of Educational Psychology, University of Nebraska-Lincoln, Lincoln, USA

**Keywords:** Development, Sleep, Circadian rhythms, Actigraphy, NPCRA (Non-Parametric Circadian Rhythm Analyses), Down syndrome

## Abstract

**Objectives:**

To evaluate sleep consolidation and circadian activity rhythms in infants and toddlers with Down syndrome (DS) under light and socially entrained conditions within a familiar setting. Given previous human and animal data suggesting intact circadian regulation of melatonin across the day and night, it was hypothesized that behavioral indices of circadian rhythmicity would likewise be intact in the sample with DS.

**Methods:**

A cross-sectional study of 66 infants and young children with DS, aged 5–67 months, and 43 typically developing age-matched controls. Sleep and measures of circadian robustness or timing were quantified using continuous in-home actigraphy recordings performed over seven days. Circadian robustness was quantified via time series analysis of rest-activity patterns. Phase markers of circadian timing were calculated alongside these values. Sleep efficiency was also estimated based on the actigraphy recordings.

**Results:**

This study provided further evidence that general sleep quality is poor in infants and toddlers with DS, a population that has sleep apnea prevalence as high as 50% during the preschool years. Despite poor sleep quality, circadian rhythm and phase were preserved in children with DS and displayed similar developmental trajectories in cross-sectional comparisons with a typically developing (TD) cohort. In line with past work, lower sleep efficiency scores were quantified in the group with DS relative to TD children. Infants born with DS exhibited the worst sleep fragmentation; however, in both groups, sleep efficiency and consolidation increased across age. Three circadian phase markers showed that 35% of the recruitment sample with DS was phase-advanced to an earlier morning schedule, suggesting significant within-group variability in the timing of their daily activity rhythms.

**Conclusions:**

Circadian rhythms of wake and sleep are robust in children born with DS. The present results suggest that sleep fragmentation and any resultant cognitive deficits are likely not confounded by corresponding deficits in circadian rhythms.

## Highlights

• Circadian activity rhythms are robust in young children with Down syndrome.
• Early morning activity is phase-advanced in a subgroup.
• Infants with Down syndrome show significant sleep fragmentation.
• Sleep efficiency improves with age in children with Down syndrome, but still lags the trajectory seen in typical development.

**Statement of Significance**

Sleep is recognized as a key component to health, yet little is understood about how sleep and its circadian timing mature during infancy or how this maturation ties into the development of brain networks underlying cognition. While previous studies have described pediatric sleep through parent and caregiver reports, new work is tracking its developmental trajectory and variations thereof, using more objective measures in typical and at-risk populations. Given the high rate of sleep problems in Down syndrome (DS) and their emergence soon after birth, the present study quantified the cross-sectional development of circadian rhythms and sleep in infants and toddlers with this genetic background. The data suggest that young children with DS maintain normal circadian timekeeping in the face of significant problems with sleep quality. Future studies will be critical towards defining the role that sleep fragmentation has in shaping cognitive outcomes across populations.

## 1 Introduction

Down syndrome (DS) emerges out of the biological sequelae produced by an extra copy of all or part of human chromosome 21 (Hsa21; trisomy-21) [1]. In addition to well-documented functional strengths, individuals with this condition face many challenges throughout their lifespan. Chief among these are mild-to-profound impairments in intellectual functioning that are reflected in and abetted by deficits in learning, memory, and receptive and expressive language [2]. Much of the cognitive profile seen in people with DS can be traced mechanistically to changes in the developmental trajectory of the frontotemporal regions of the brain, including the hippocampus. Neuropsychological research has supported this relationship by repeatedly documenting a disproportionate weakness in performance on cognitive tasks that are dependent on hippocampal function in those with DS [1], [2], [3].

Alongside differences in brain architecture and connectivity are several other medical comorbidities with the potential to limit how successfully the brain of a person with DS is able to recapitulate typical development. Infants with DS are born with smaller neurocraniums and experience changes to bone growth along the craniofacial skeleton that physically compress the midface and jaws [4]. Soft tissue crowding of the pharynx and palate ensues, which is exacerbated by a posterior displacement of the tongue, enlarged tonsils and adenoids, and medially displaced tonsils that occlude the back of the throat [5], [6], [7]. These craniofacial features ultimately narrow airflow passage through the upper respiratory tract and, together with decreased pharyngeal muscle tone and airway collapse, result in obstructive sleep apnea syndrome (OSAS) and sleep fragmentation in the majority of individuals with DS [8], [9], [10], [11], [12], [13].

The high prevalence of OSAS in people with DS has been noted by several physicians over the past three decades (eg, Southall, Marcus, Schott, et al. [8], [9], [10], [11], [12], [13] ) and has shaped guidelines for the supervision and care of children with DS. For instance, the American Academy of Pediatrics recommends that children born with DS should receive monitoring from birth and a polysomnogram (PSG)-measured sleep assessment by four years of age [14]. However, a synthesis of recent work has suggested that younger children might benefit from earlier efforts to actively detect (and treat) OSAS. At least two studies have screened infants with DS between 1 and 40 weeks of age for sleep-related upper airway obstruction [13], [15]. In one of these studies, the authors used a cohort of children with a mean age of 44 days [15]. The aggregated data suggest that symptoms of OSAS (oxygen desaturation with continued attempts at breathing, elevated capnography readings, apnea-hypopnea index (AHI) levels >5) are not only present in *at least* 30–50% of infants with DS, but that when present, often meet criteria for severe OSAS (defined by AHI levels >10) [13], [15].

Upper airway obstruction and general neurological delay likely lead to disturbed sleep in younger children with DS. Several investigations using short-term and longitudinal electroencephalogram (EEG) analysis have noted that: (1) these children arouse more during nighttime sleep than typically developing, chronological age-matched controls, (2) spend less time in deeper stages of non-rapid eye movement (NREM) and rapid eye movement (REM) sleep, (3) shift more often from deeper NREM stages of sleep to lighter ones, and (4) exhibit less spindle activity [16], [17], [18], [19], [20]. Sleep-disordered breathing and poor sleep quality in the pediatric population with DS have been associated with restless nighttime movement and parasomnias, sleep anxiety, daytime fatigue, and decreased scores on inventories of adaptive function like the Life Habit Questionnaire (Life-H) [21], [22], [23], [24]. These findings suggest that poor sleep might have real-world consequences for the functional daily living and school performance of the average child with DS. Mounting evidence also suggests that poor sleep might negatively impact cognitive development in these children.

Although the notion has received very little empirical study, it is reasonable to assume that sleep in early life plays a formative role in setting up typical and atypical cognitive systems. Down syndrome provides a unique model of exaggerated sleep disruption during the infant and toddler period, at a time when the brain's frontotemporal cortices are developing rapidly to support the establishment of cognitive precursors to domains like executive function (EF) or language, and their maturation into adult forms within efficient brain networks. The first group to report links between subjective ratings of OSAS and EF in the adolescent and young adult DS population was led by Chen et al. [25]. The researchers found that those with DS who had more severe behavioral symptoms of OSAS were impaired on verbal fluency, rule-shifting, and behavioral inhibition tasks relative to chronological and mental age-matched individuals with DS who had fewer OSAS symptoms.

To track the origin of these deficits, Edgin et al. looked at two progressively younger cohorts of children with DS, one 7–12 years of age, the other 2–5 years [26], [27]. Between 7 and 12 years, children with DS comorbid for OSAS (ie, AHI levels >1.5, as determined by ambulatory PSG) performed significantly worse on an EF set-shifting task and had verbal IQ scores almost a standard deviation (ie, nine points) lower than a similarly comprised group with DS not meeting criteria for OSAS. In that study, differences in EF and language could not be attributed to body mass index (BMI), daytime sleepiness, or difficulties with maintaining attention [26].

Extending these observations, the researchers then examined how disrupted sleep might relate to language development in toddlers with DS (2–5 years) compared with typically developing (TD) children. Five consecutive days of actigraphy were collected alongside 16 continuous hours of auditory recordings with a Language Environment Analysis (LENA) monitor. Actigraphy revealed that the majority of the DS cohort, upwards of two-thirds, had average sleep efficiency scores <80% (ie, were poor sleepers), compared with 15% of the TD sample [27]. Analysis of the sound environment suggested that this disparity in sleep quality influenced precursors to language output; the mean length of utterance for poor-sleeping children with DS was significantly shorter than for children that slept well (although utterances for both groups with DS were truncated relative to TD children). Degree of sleep consolidation also correlated with how well the children with DS were able to combine words and form sentences on the MacArthur-Bates Communicative Development Inventory (MB-CDI) [27]. It is noteworthy that these results were robust when controlling for medical and social background factors and for behavioral measures associated with autism spectrum symptoms.

Overall, the findings from Edgin et al. have established that some variation seen in younger and older children with DS on EF, verbal IQ, and language ability is related to sleep quality [25], [26], [27]. Perturbations in sleep might negatively impact development of the frontal and medial temporal lobes in people with DS, placing functions associated with these areas of the brain at greater risk. While these findings suggest tangible treatment solutions for improving cognition in the pediatric population with DS via sleep intervention, they are tempered by a lack of certainty as to the origin of the sleep problems. Do these deficits occur solely as a result of OSAS and the accompanying fragmentation of the sleep period, or are they also a by-product of circadian rhythm dysfunction? Many aspects of sleep are determined by an interaction between homeostatic and circadian regulatory processes that balance the need for sleep versus its timing during the 24-h cycle [28]. The possibility remains that sleep disruption in children with DS occurs because the circadian system is not sending a clear signal at night to drive down arousal and maintain sleep.

In an effort to answer this question, the present study assessed sleep, with seven days of actigraphy, and quantified objective behavioral measures of sleep efficiency and circadian prominence in infants and toddlers with DS relative to an age-matched TD control group. These non-invasive recordings were conducted in the home, as the children went about their regular day-to-day activities, and did not disturb their daily routines. The study reported here is the first to characterize the circadian activity profile of children with DS over an extended period of sleep measurement, and is the largest cross-sectional actigraphy analysis ever conducted in this population.

## 2 Methods

### 2.1 Participants

A community-based sample of 77 children with DS and 53 TD controls was recruited between 2011 and 2015 from an investigation of sleep and learning conducted by the Memory Development and Disorders Laboratory at the University of Arizona (MDDL-UA). Participants with or without DS were recruited through advertisements in local and national news venues, including community events, newsletters, research registries, word of mouth, and social media. In all cases, the same marketing communication materials were used. Two additional recruitment mechanisms were used to enroll children with DS that were not employed in TD children. Enrollment of the group with DS was bolstered by study advertisements that were distributed through the National Institutes of Health National Down Syndrome Registry (DS-Connect^®^) and via outreach events in the local DS community, such as the Tucson Buddy Walk. A majority of the TD sample and the sample with DS resided in the state of Arizona (80/130), with others located across the United States of America (USA).

In the current study, seven days of home-based actigraphy data and parent reports on sleep were collected. Parents were required to maintain a sleep diary and provide responses on select items from the Children's Sleep Habits Questionnaire (CSHQ), a pediatric assessment that screens for symptoms of sleep problems defined in the International Classification of Sleep Disorders (ICSD-2) diagnostic and classification manual [29]. The CSHQ has been validated in TD children aged 2–10 years [30] and in children from diverse neurodevelopmental backgrounds such as autism and DS [23], [30], [31]. Because the instrument has not been used to characterize sleep habits in infants and younger toddlers between the ages of 5–23 months, parents were asked to only address questions on the CSHQ that could be directly related to variables from the actigraphy recordings and sleep diary.

Patient medical records were faxed or physically mailed on hardcopy or CD to the MDDL-UA. A diagnosis of trisomy-21 was confirmed by karyotype for those in the group with DS. One child included in the group with DS was determined to have translocation DS (ie, with an extra copy of Hsa21 physically attached to another chromosome). Although none were recruited for study, no effort was made to specifically exclude children with mosaic DS (ie, children with a mix of cells that either have or do not have an extra freely segregating copy of Hsa21). Written consent was obtained from the parents or legal guardians of the participants before assessment, and the UA Institutional Review Board approved all the procedures. All participants were too young to consent; thus, verbal assent was obtained.

**Fig. 1:**
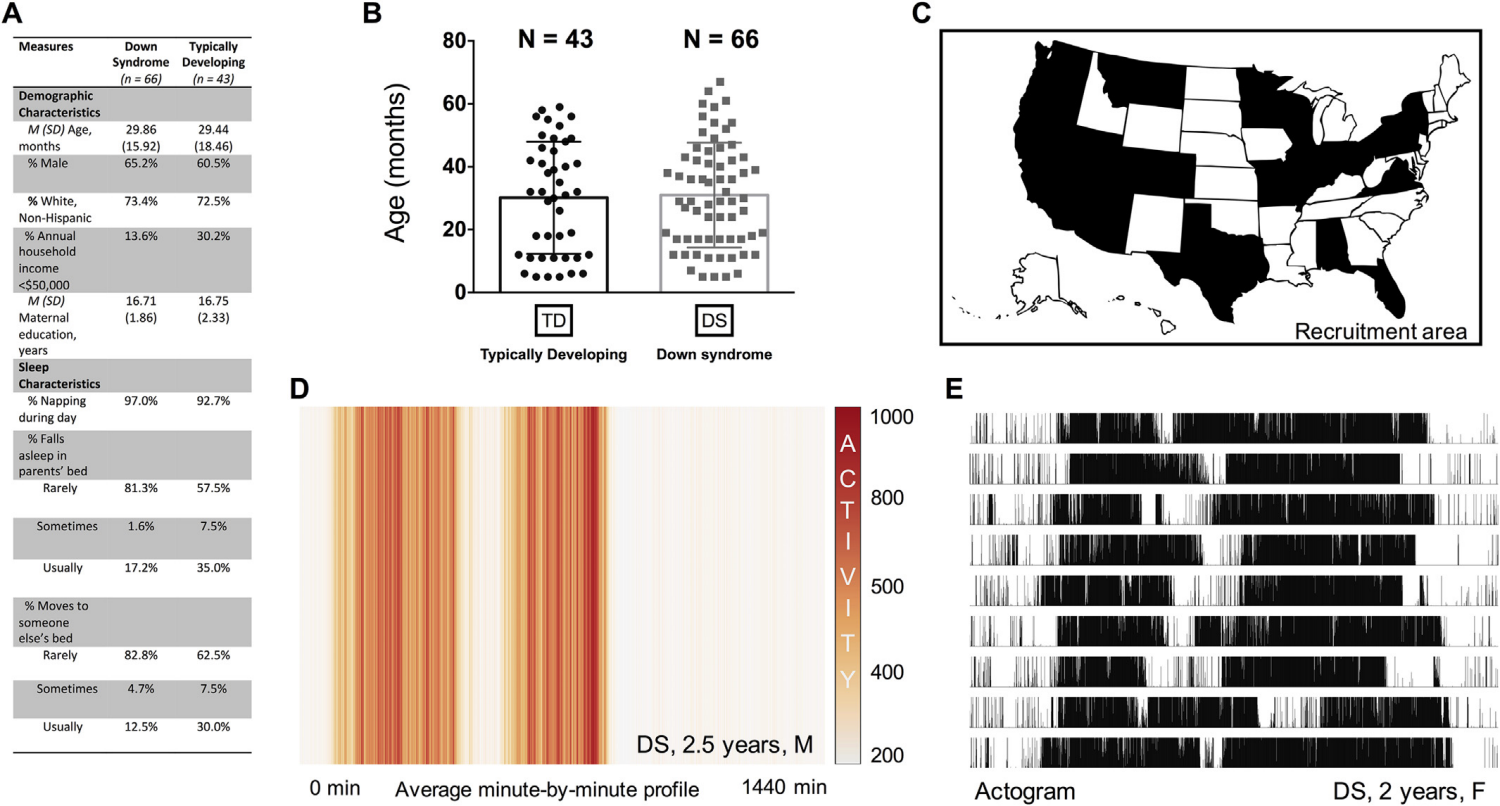
**A and B**. The Down syndrome (DS) and typically developing (TD) groups were equivalent along several demographic and social background factors, and exhibited a similar age distribution. Categories listed under sleep characteristics were curated from questions on the Children's Sleep Habits Questionnaire. Based on these questions, children from the DS sample were found to co-sleep less with parents or siblings during nighttime rest than those from the TD sample. Nearly all the children in the survey (>90%), irrespective of genetic background, were classified as habitual nappers. (**C**) Map of the recruitment area. States in black mark where the participants lived at the time of the actigraphy recordings. (**D**) Heat map of a minute-by-minute activity profile generated from several days of recording from a 2.5-year-old male toddler with DS. Lack of color diffusion in the onset and offset areas suggests that the movement of this individual was confined to two blocks of activity occurring at approximately the same time each day, separated by a routine nap. (**E**) High-resolution actogram showing the absolute activity or inactivity of a 2-year-old female toddler with DS. Data are vertically aligned; such that one 24-h day of movement is shown per line, with successive days appearing one below the other. Any movement registered with the Actiwatch, no matter the intensity, was tallied with a black tick. White space indicates complete inactivity. The graph suggests that the child had robust circadian patterns of behavior.

Exclusion criteria for sleep and circadian analyses included the following: (a) <5 full consecutive days of actigraphy (*n* = 14); (b) parents indicated that the child was sick during the recording period (*n* = 2); (c) parents indicated traveling out of state during the recording period (*n* = 1); (d) actigraphy was performed within a 10-day window following a daylight savings change (*n* = 1); or (e) gestational age <36 weeks (*n* = 3). These filters resulted in a final sample of 66 children with DS and 43 TD children, with 70 individuals residing in Arizona (Fig. 1). The participants ranged in age from 5 to 67 months in the sample with DS (M [SD] age = 29.86 [15.92] months, 43 males and 23 females) and, similarly, from 5 to 58 months in the TD sample (M [SD] age = 29.44 [18.46] months, 26 males and 17 females) (Fig. 1A and B). For the two groups, there were no significant differences observed in the average or relative distributions of their age, gender, race/ethnicity, maternal education, or annual household income (*t*-test, Levene's, Fisher's exact, or Chi-squared tests, all *p* > 0.05; Fig. 1A). The percentage of nappers was also tightly conserved between the two: almost all the children included in the present study (>90%) napped during the day, whether they had DS or not. However, based on parent answers to select questions on the CSHQ, TD children were significantly more likely to co-sleep with parents and siblings at night than the children in the cohort with DS (Χ^2^ = 6.17–7.75, *p* < 0.05; Fig. 1A).

### 2.2 Actigraphy: assessment of circadian rhythms

Actigraphy is particularly well suited for examining circadian rhythms in young children and those with intellectual disabilities, given the ease of data collection over multiple days. Study equipment was shipped by courier or hand-delivered to the participants' homes. Parents were instructed to place the Actiwatch on their child's non-dominant wrist, or in the case of infants, on the infant's non-dominant ankle, for a minimum of seven consecutive days, and were informed that the device could be worn during bath time and if in the pool for <30 min. Data were collected using an Actiwatch-2 (weight = 16 g, Philips Respironics USA, Koninklijke Philips N.V.), which is a solid-state piezoelectric monitor that converts acceleration-mediated changes in voltage into discrete signals indicating movement. The sample rate of the Actiwatch was set at 32 Hz and the peak range of sensitivity was 0.5–2 G. All data were summed and collected in 30-s epochs.

Unparsed Actiware files containing 2880 points of data/24 h were plotted as a time series and analyzed using ClockLab for a quantitative description of diurnal rhythms in the TD group and in the group with DS (Actimetrics Version 6, Wilmette, IL, USA). The actogram window was modified so that only full-day periods were seen and used in the analyses. The first set of measures that were estimated were phase markers associated with circadian timing and their corresponding variations. Daily onsets (the time of day when an individual wakes) were calculated using a template-matching algorithm that searched for a time point at the intersection of a 5-h period of relative immobility followed by a 5-h period of vigorous movement. Daily offsets (the time of day when an individual falls asleep) were determined using a reciprocal 5-h template-matching strategy. Daily acrophases (the time of day when an individual is most physically active) were calculated by using the least squares method to fit each day's activity profile to a 24-h sine function and quantifying the temporal position of the resulting sine wave's peak. The individual values for these three circadian phase markers were adjusted to account for time zone differences and manually inspected and corroborated by a second investigator to ensure accuracy.

To obtain estimates of robustness for the circadian component of the actigraphy data, Lomb-Scargle Periodogram analysis (LSP) and a classic Fast-Fourier transform (FFT) were used. The power spectrum (amplitude) from 18 to 30 h was determined for each. Lomb-Scargle Periodogram is a particularly useful method for detecting periodicities from incomplete, evenly sampled time series because it can process records with large or frequently reoccurring gaps of 1–2 h that might happen at a similar time each day [32], [33], [34]. The fact that it can avoid inflating estimates of circadian rhythmicity in an actigraphy data set when gaps are present at regular intervals is important for assessing pediatric activity records, which often contain nap periods that are routinely scheduled at particular points during the afternoon.

Non-Parametric Circadian Rhythm Analyses (NPCRA) of interdaily stability (IS), intradaily variability (IV), and relative amplitude (RA) were also used to assess robustness in the circadian range for both the TD group and DS group. A mathematical description of these variables can be found in Van Someren et al. [35]. In healthy subjects, the activity profiles that are recorded from one day to the next will resemble each other because the person's endogenous circadian clock is phase-locked to stable environmental cues that occur over a 24-h cycle, primarily variations in light intensity pursuant to the solar light–dark cycle. The IS value gives an indication of how well a person's rest-activity patterns are maintained across days and weeks, and quantifies the strength of their coupling to the environment. Interdaily stability values range between 0, indicating pure Gaussian noise, to 1.0, which means that the 24-h activity profile is recapitulated perfectly every single day [35], [36], [37], [38].

In healthy subjects, periods of rest and activity will also tend to be consolidated to one or two major episodes within a day. Those with circadian disorders might experience more erratic changes in arousal that appear as activity fluctuations in the actigraphy record. The IV value gives an indication as to how often these low-activity/high-activity transitions occur, and quantifies the degree to which behavioral rhythms are fragmented. Generally, IV values range near 0, indicating that transitions occurring between rest/activity within a day can be described by a perfect sine wave and are tightly consolidated (occurring almost digitally), to 2.0, which means that the transitions are fragmented to the point of being random [35], [36], [37], [38].

The last NPCRA variable calculated in the circadian analysis, RA, is a non-parametric value that has historically been used to approximate the robustness of a circadian rhythm [35], [36], [37], [38]. It compares the normalized difference in magnitude between the most active 10-h period of the day (M10) to the least active 5-h period (L5), with a range of 0–1 (higher values represent a greater divergence between the two phases).

### 2.3 Actigraphy: assessment of sleep

In several independent and meta-analyses, actigraphy has been found to correlate significantly with PSG in measurement of total sleep time and efficiency, although it fares less well in detecting wake [39], [40], [41], [42]. It predicts sleep behavior in infants when directly compared to PSG [43] and has been used in several pediatric populations for the study of sleep-circadian disorders [44], including studies conducted in people with DS [45], [46]. Sleep variables were assessed at the medium sensitivity threshold (40 counts/epoch) and analyzed with Actiware software 6.08 (Philips Respironics, USA, Koninklijke Philips N.V.). The built-in Actiware software thresholds were used because no algorithms have been developed at this sampling rate for infants with extensive sleep impairment (as is found in infants and toddlers with DS). When comparing PSG-measured sleep to actigraphy in toddlers with DS, it was found that the actigraphy-derived sleep efficiency variable correlates well with PSG-measured EEG arousals (*n* = 18; *r* = −0.71, *p* < 0.05; unpublished observation). Previous work has also shown that the prevalence of periodic limb movement disorder (PLMD) among children with DS is comparable to the prevalence observed in the TD pediatric population (ie, about 20%) [47]. These estimates suggest that involuntary movements at night are unlikely to disproportionately influence the actigraphy recordings of the group with DS versus the TD group. The estimates place limits on the possibility that PLMD symptoms might contribute to false positive readings of low sleep efficiency in children with DS.

Using Actiware, sleep onset was marked by a period of ≥3 min of immobility, while sleep offset was marked by a period of ≥5 min of continuous movement once a sleep episode had started. The two markers provided an estimate of sleep duration. During behaviorally defined sleep, individual 30-s epochs were classified as “wake” or “sleep” based on a weighted sum of the activity in that epoch versus the activity in a time window of a few minutes bookending it. If the sum exceeded a particular threshold, the epoch was considered one in which the subject was awake. Based on this criterion, each full night's sleep period was scored as a sequence of sleep/wake epochs from which sleep efficiency (ie, percentage of epochs scored as “sleep” versus “wake” from sleep onset to offset) could be derived. Caregivers also completed a seven-day sleep diary, which was necessary to determine the location onsets and offsets of nighttime sleep and provided a means by which to evaluate any discrepancies that cropped up with the actigraphy records. By and large, the onsets and offsets computed from the actigraphy data showed agreement with parental self-reported wake-up times and bedtimes.

### 2.4 Statistical comparisons

All statistical operations were carried out in SPSS 23.0 (IBM Corp., Armonk, NY, USA). Tests for normality conducted prior to analyses revealed significant skewness and kurtosis for most measures associated with circadian timing and robustness, leading to a logarithmic transformation of these data. Following the calculation of descriptive statistics, a series of hierarchical linear regression models were constructed. The dependent variables – sleep efficiency and duration, phase onsets, offsets, and acrophases, the amplitudes of the 24-h periodicities in the LSP and FFT, and NPCRA measures IS, IV, RA, M10, and L5 – were regressed on the independent variables of group (DS versus TD), age, and interactions of group X age. For all cases, group was coded as 1 for DS and 0 for TD, with age measured in months. Interaction terms were constructed by multiplying these predictors. A forward stepwise modeling approach was used, with main effects of group and age entered first and interactions added in the second phase of model building. If the *p*-value for the interaction term exceeded 0.10, this term was backward trimmed from the model, in the interests of parsimony. Two-tailed tests were adopted throughout, with alpha set at >0.05.

## 3 Results

### 3.1 Prominence of circadian rhythms and sleep

**Fig. 2:**
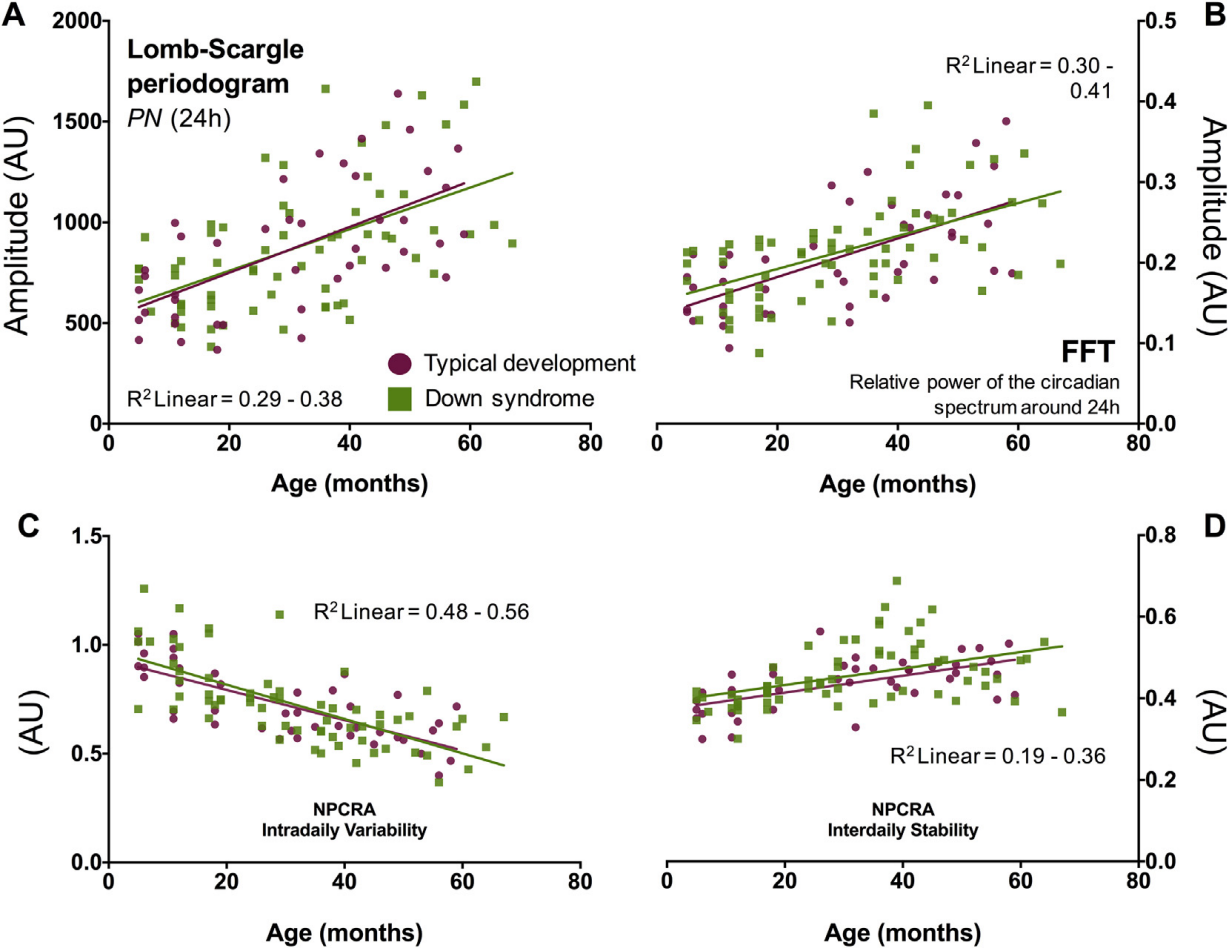
**A–D**. Quantitative assessment of circadian robustness in the Down syndrome (DS) (green squares) and typically developing (TD) (purple circles) groups under entrained conditions. Raw values for LSP_24h_, FFT_24h_, IV, and IS are plotted for each study participant as a function of age. The goodness-of-fit (R^2^ linear) and slope of the regression lines fitted to each panel suggest that circadian rhythms of behavioral activity mature equally well in children with and without DS from 6 months to 5 years. PN, dominant frequency in the Lomb-Scargle periodogram; FFT, fast Fourier transform; NPCRA, Non-parametric circadian rhythm analysis; IV, Intradaily variability; IS, Interdaily stability; AU, arbitrary units. (For interpretation of the references to colour in this figure legend, the reader is referred to the web version of this article.)

The descriptive statistics and regression models for all the sleep-circadian variables are summarized in Table 1, Table 2. Representative actograms are provided in Fig. 1D and E. Children with DS showed no differences in the LSP_24h_ or FFT_24h_ amplitude relative to the TD group (Table 1, Table 2; Supplemental information). However, age did explain about 33% of the variance for each measure (*p* < 0.001; Table 2, Fig. 2A and B). For instance, a one-month increase in age from 0 to 67 months was associated with a ∼7-point increase in LSP_24h_ amplitude in both the DS and TD samples (β = 0.57, *p* < 0.001). Interactions between group and age were not significant in any of the LSP or FFT regression models.

In addition, NPCRA-IV and NPCRA–IS values bore a strong relation to age, but did not distinguish children born with DS from those with a typical genetic background (*p* > 0.70 and 0.13, respectively; Table 1, Table 2). Increasing age from 0 to 67 months predicted lower IV, indicating that older children, irrespective of trisomy, showed less waxing and waning of behavioral activity within the 24-h day and more wake/sleep consolidation than younger children (Fig. 2C). Increasing age also signaled higher IS values in both the TD group and the group with DS (Fig. 2D); presumably, older children are better synchronized to the solar cycle and exposed to more social cues as they enter their preschool years than homebound infants or toddlers. As with the LSP and FFT variables, group X age interactions were not significant in the NPCRA regression models.

**Fig. 3:**
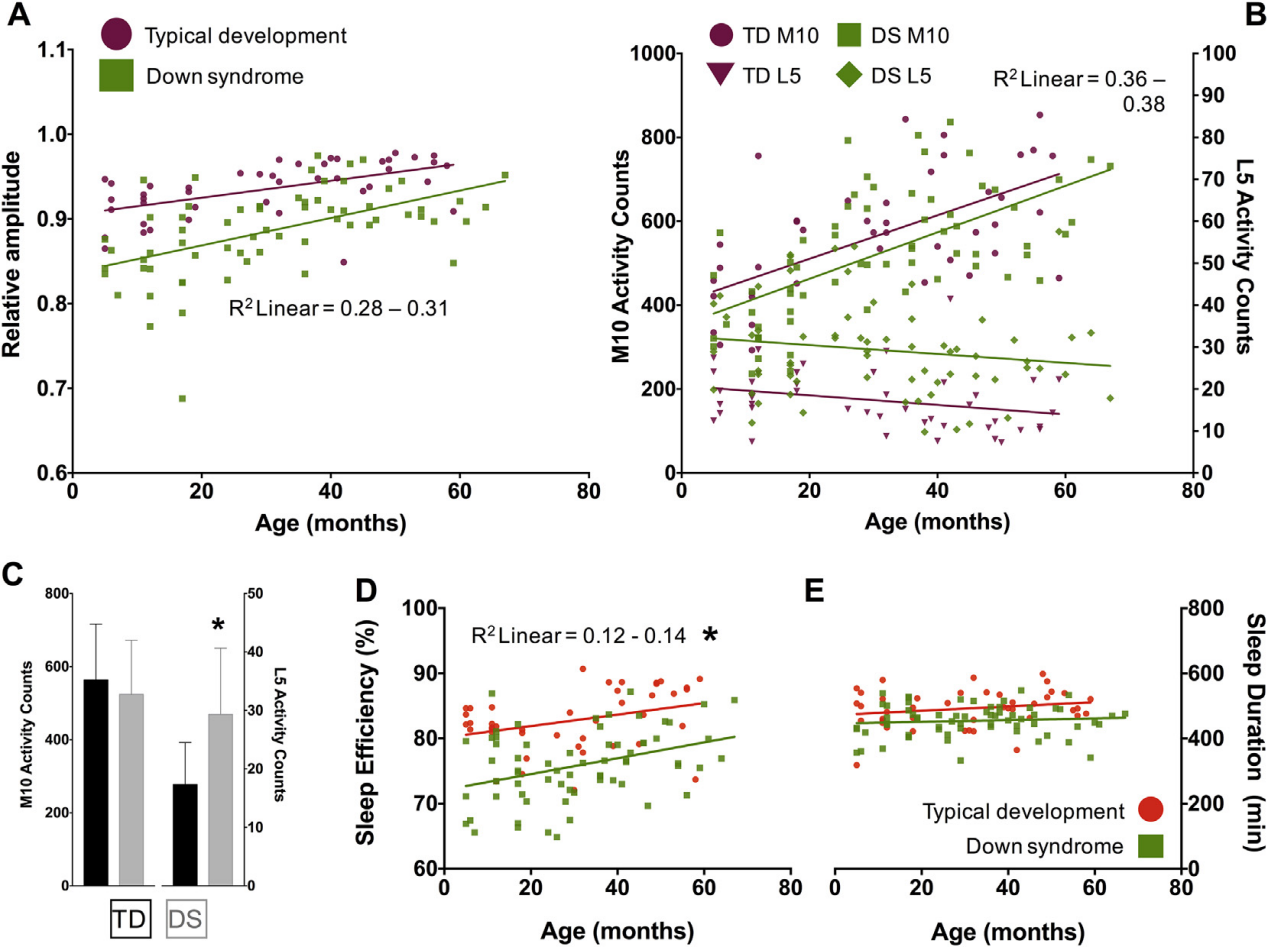
**A and B**. Raw NPCRA-RA values, and raw values for the RA components M10 and L5, are plotted for each study participant as a function of age (TD RA = purple circles; TD M10 = purple circles; TD L5 = upside down triangles; DS RA = green squares; DS M10 = green squares; DS L5 = green diamonds). The goodness-of-fit and slope of the regression lines fitted to the RA scatter plot suggest that RA increases at a similar rate in both the Down syndrome (DS) and typically developing (TD) groups. However, children with DS have smaller RAs compared with children without DS matched for developmental time point. **B and C**. RA reductions in the Down syndrome (DS) group appear to result from differences in L5, but not M10. M10 and L5 values change in a similar fashion across age in both groups, but L5 values are up-shifted in infants, toddlers, and school-aged children with DS compared with their typically developing (TD) peers. **D and E**. Raw values for sleep efficiency and duration are plotted for each study participant as a function of age (typically developing (TD) = orange circles; Down syndrome (DS) = green squares). Sleep is better consolidated over the course of development in both the DS and TD groups, but its efficiency is significantly lower in children with DS. Sleep duration does not change from 6 months to 5 years, but is also lower in the DS group by a small, but significant, margin. NPCRA, Non-parametric circadian rhythm analysis; RA, relative amplitude; M10, Most active 10-h period of the day; L5, Least active 5-h period of the day. (For interpretation of the references to colour in this figure legend, the reader is referred to the web version of this article.)

As reported in Table 1, Table 2 and illustrated in Fig. 3A, NPCRA-RA values were significantly lower in the sample with DS versus the TD sample, but generally increased with age (*p* < 0.001; Supplemental information). To understand the factors that were driving this amplitude reduction in the sample with DS, the M10 and L5 values were compared from both groups (Table 1, Table 2, Fig. 3B and C). With greater age, children born with DS or those from a typical background saw similar decreases in movement during the least active parts of the day (L5 activity, *p* = 0.019) and similar increases in movement during the most active parts of the day (M10 activity, *p* < 0.001; Table 2, Fig. 3B). However, there was a dichotomy with regards to group differences on each measure. While M10 values were not statistically different between the TD sample and the sample with DS (*p* > 0.05 in regression model, *p* = 0.184–0.187 in group-wise comparisons with two-tailed *t*-tests; Supplemental information), L5 values were (Fig. 3B). Children with DS were almost twice as active during L5 compared with typical children (Table 1, Table 2; Fig. 3B and C; *p* < 0.001), irrespective of age; no significant interactions between age and group were found for any of the RA, M10, or L5 regression models.

The L5 period that was estimated in ClockLab invariably coincided with the sleep period that was registered with the Actiwatch Actiware software, raising the possibility that the increase in L5 activity and decrease in NPCRA-RA observed in the cohort with DS related to group differences in sleep fragmentation. Consistent with this interpretation, children with DS exhibited an average sleep efficiency that was 7% lower than TD controls over the several days of Actiwatch recording (Table 1, Table 2, *p* < 0.001). While each one-month increase in age predicted a 0.8% increase in sleep efficiency in both the TD group and the group with DS (*p* < 0.01), the interaction between group and age was not significant (*p* = 0.13), meaning that the negative effect of the DS genetic background on sleep efficiency, like its effects on L5, did not vary by age (Fig. 3D).

Finally, participants in the group with DS slept on average 39 min less than the TD sample (β = −0.34, *p* < 0.001, Model R^2^ = 0.13; Table 1 and Fig. 3E). There were no correlations between sleep duration and age (*p* = 0.139) or interactions between these predictors (*p* = 0.497).

### 3.2 Phase markers of circadian timing: chronotypes

**Fig. 4:**
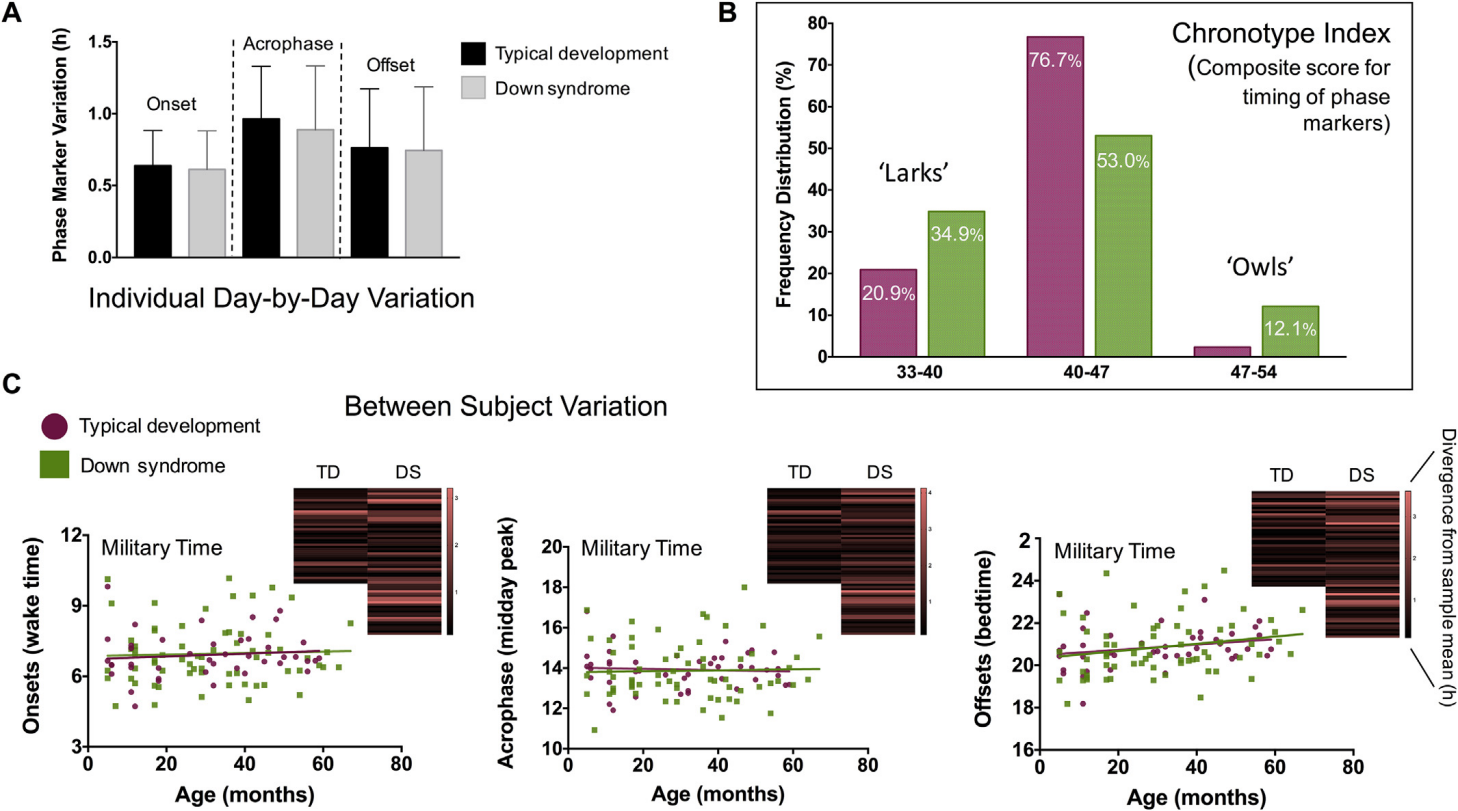
**A**. Bar graph showing the variation exhibited in an individual's phase marker timing averaged within the Down syndrome (DS) and typically developing (TD) groups (onset = the time of day when person awakes; acrophase = time of day when person is most physically active; offset = time of day when person falls asleep; SD = standard deviation). Relative to the TD sample, young children with DS exhibit no evidence of circadian dispersion in the times of day when they wake-up, are most highly active, or fall asleep. In both groups, the schedule of these events deviated by 30–60 min from one day to the next during the actigraphy recording period. **B and C**. The average onset (lower left panel), acrophase (lower middle panel), and offset (lower right panel) times displayed by each study participant is plotted against age. These phase markers do not change appreciably over the course of development, but are more widely distributed in children with Down syndrome (DS) compared with typically developing (TD) controls. In particular, more children with DS than TD have exaggerated chronotypes, where their activity is biased towards either earlier-than-average parts of the day (ie, larks; extreme-morning people) or later-than-average parts of the day (ie, owls; evening-type people) (TD = purple circles and bar graphs; DS = green squares and bar graphs). Heat map inserts provide an alternative visualization to this chronotype spread; the divergence from the sample mean of each study participant's average “stable” onset, acrophase, and offset time is color coded so that red lines indicate subjects with phase marker values further away from the group average. DS heat maps appear more in red than TD ones. (For interpretation of the references to colour in this figure legend, the reader is referred to the web version of this article.)

Hierarchical linear regression models using log-transformed numbers and group averages of raw data suggested that trisomy-21 did not impact most markers of circadian timing or their within-subjects variation (eg, how consistently one wakes up at the same time each day over the actigraphy monitoring period) (Table 1, Table 2; Fig. 4A; Supplemental information). Out of the six measures associated with circadian timing, none were influenced by genetic background, and only offsets and onset variations significantly correlated with age. Each 1-month increase in age was associated with: (1) a 0.18-min increase in the time the study participants went to bed (β = 0.22, *p* < 0.05; Table 2 and Fig. 4C, right panel); and (2) a 10-min decrease in the variability with which they woke up (β = −0.22, *p* < 0.05).

While plotting the average onset, acrophase, and offset times exhibited by each participant in the sample pool over the recording period, it was noticed that the spread of values or “chronotypes” was much wider among the subjects with DS than for the typically developing subjects (Fig. 4B and C, all panels). Levene's test for equality of variances on the log-transformed numbers indicated that the standard deviation for these phase markers was statistically different between the TD group and the DS group (*p* ≤ 0.05; Table 1; Supplemental information), but for no other measures save for NPCRA-RA. People's endogenous circadian clocks will “phase-lock” differently to the light–dark cycle. Some clocks are phase advanced in their synchronization, implying that a person is waking up before the sun rises and falling asleep before the sun has set (or in both cases, soon thereafter) [48], [49]. This chronotype is referred to as a “lark”. Other clocks are phase delayed in their synchronization, implying that a person is waking up after the sun has risen and falling asleep after the sun has set (or in some cases, well after dawn or dusk) [48], [49]. This chronotype is referred to as an “owl”. Here, the data suggest that individuals with DS vary more dramatically in their distribution of larks and owls than the TD population across early childhood (Fig. 4C, along with heat map inserts).

**Fig. 5:**
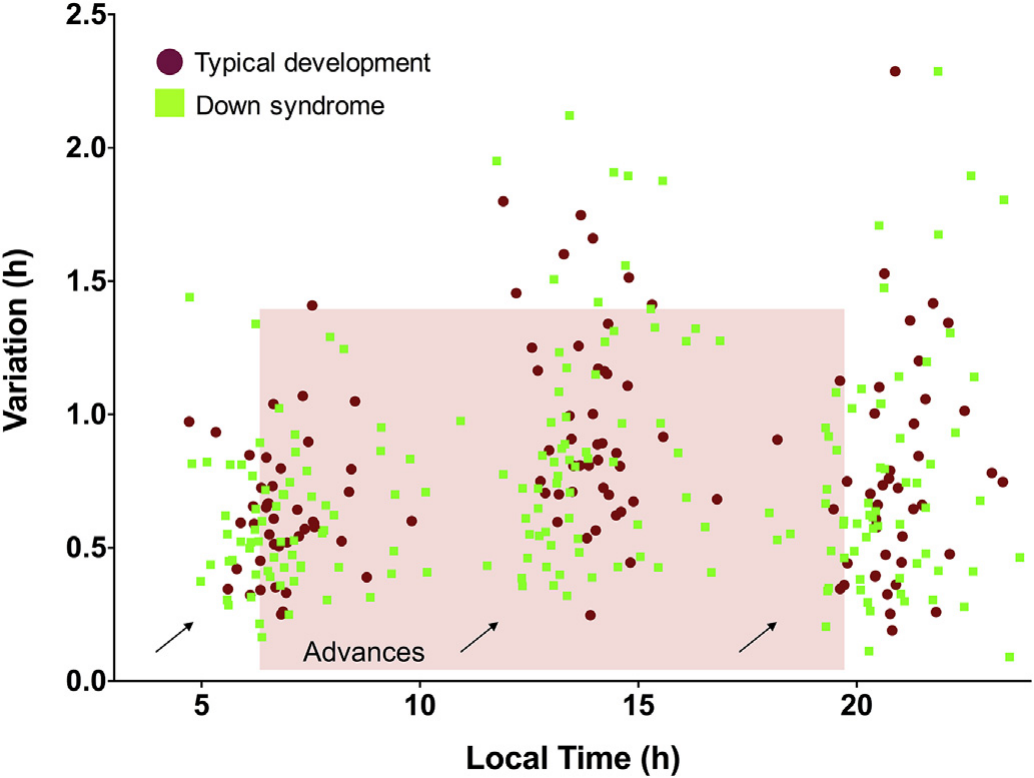
The average onset, acrophase, and offset times calculated for each subject in the Down syndrome (DS) (green squares) and typically developing (TD) (red circles) groups are plotted along the *x*-axis and organized according to their intra-individual day-to-day variation (*y*-axis). These data suggest that the DS “lark” chronotype subgroup (indicated by arrows in the lower left of the morning, afternoon, and evening cluster of data points) is particularly stable relative to other chronotypes observed in TD children or those with DS. (For interpretation of the references to colour in this figure legend, the reader is referred to the web version of this article.)

To get a sense for whether this change in chronotype distribution between the two groups was significant, a chronotype index was created for each child under study by adding the total hours from midnight (ie, ZT 0) that their average onset, acrophase, and offset occurred. If, on average, a child woke up at 07:00 (+7 h), was most highly active at 13:00 (+13 h), and then went to bed at 20:00 (+20 h), they would accrue a chronotype index of 40. Next, these data were organized into three evenly spaced bins, corresponding to larks (composite scores 33–40), owls (composite scores 47–54), or those falling in between (composite scores 40–47). Consistent with previous results, it was found that many young children – irrespective of genetic background – demonstrated early chronotypes. However, on a relative percentage basis, more infants and toddlers with DS were classified as larks than TD children (Χ^2^ = 7.09, *p* = 0.029; Fig. 4B). In addition, an appreciable number, 12.1%, showed late chronotype features that went largely unseen in the TD group (Fig. 4B). The salient shift in children with DS to earlier chronotypes is illustrated in Fig. 5; the average value for each phase marker is plotted as a function of its intra-individual variation. This visualization suggests that “larks” with DS were among the most stable chronotypes observed during the actigraphy recording period.

## 4 Discussion

In the aggregate, the cross-sectional data suggest that children with DS see a remarkable conservation in the development of their circadian rhythms that parallel the developmental course seen in children from a typical genetic background. As they move from infancy to middle childhood, individuals with DS exhibit a similar strengthening in the 24-h pattern of their behavioral activity, consolidate their activity and rest at more discrete periods of the day, and preserve this 24-h schedule better across time. These data complement findings that have accumulated over several studies looking at the circadian function of two independently derived mouse models of DS. Adult mice engineered to overexpress various ensembles of genes that are triplicated in people with DS showed normal 24-h variations of locomotor activity and wheel-running in their home cages [50], [51], [52], along with proper circadian responses to different lighting conditions [50], despite differences in sleep architecture from wild-type littermates [52], [53], [54]. The behavioral readouts of circadian organization reported here for people with DS and in the aforementioned mouse models fit nicely with previous work demonstrating that day–night patterns of melatonin secretion remain tightly coordinated in both species [55], [56].

The circadian system emerges largely intact in those with DS, but the present sample of children with DS across the USA also suggests that the circadian clock in people with DS can adopt a wider variety of phase relationships with the solar cycle. In particular, a significant number of individuals with DS from the present cohort were consistently phase-advanced, relative to TD controls. While the functional significance of this lark chronotype shift has yet to be examined directly in the population of those with DS, some evidence hints at the possibility that it could materialize in performance differences across the day in school-aged children with DS. Ashworth et al. recently studied the ability of children with DS to remember pseudo-words artificially paired to well-known animals (eg, Basco = cat) [57]. They trained 6–12 year olds with DS during the morning or evening on these word-animal associations and tested recall in 24-h increments thereafter. The researchers found that the group with DS continued to improve their learning over the retesting interval if the children were originally trained and tested in the morning, but not if this instruction was given later in the day [57]. As observed in typical aging individuals [58], [59], [60], [61], the chronotype as well as performance curves for younger individuals with DS appear to be shifted to earlier times of day, although further research is necessary to causally relate the two in people with DS and better define the magnitude of chronotype differences between the population with DS and the TD population. This latter point is especially relevant, given the possibility that recruitment efforts for sleep-circadian studies, in general, might inadvertently attract children with extreme chronotypes.

Unlike circadian function, nighttime sleep consolidation was impaired in infants and toddlers with DS, relative to an age-matched and demographically matched group of TD children. The first indication of fragmented sleep was unexpectedly observed in the NPCRA-RA values of those in the group with DS. This measure has historically been used to quantify circadian robustness [35], [36], [37], [38], but here, was contaminated in the DS sample by levels of poor sleep efficiency and nighttime unrest that inflated L5 activity during the actigraphy recording period (ie, without a corresponding flattening of M10 activity). The present results suggest that the NPCRA-RA index needs to be interpreted cautiously in future circadian assessments, because the measure can be influenced by how well sleep is maintained over the evening, not just by whether there are bona fide 24-h variations to movement.

Significant sleep fragmentation was also documented in the DS cohort by Actiware analysis, which indicated that sleep was disturbed from the earliest surveyed developmental time points and, in fact, was most disturbed in infants (Fig. 3D). Previous studies have quantified sleep efficiency measures in an older pediatric sample with DS with activity-monitoring devices like those used in the present work. In line with the present data (Table 1), these studies estimated a similar 5–7% efficiency gap in the nocturnal sleep of older children with DS versus TD children [45], [46]. What is concerning about these collated observations is the fact that some degree of sleep deficit is likely to factor into the brain development of the majority of individuals with DS. The consequences of the impairment are unknown, though it is a reasonable hypothesis to suggest that poor sleep contributes to the severity and profile of intellectual disability seen in people with DS. Language achievements might be pointedly affected, given that sleep appears most fragmented from 0 to 36 months (Fig. 3D) – an age range where many developmental milestones of language learning are met [62].

Fragmented sleep in children with DS has implications for brain development and the progression in early life of what have been historically viewed as “aging” phenotypes related to Alzheimer's disease (AD). By virtue of increased dosage and metabolism of the Hsa21 gene product, amyloid precursor protein (APP), people with DS will show neurohistopathological hallmarks of AD by as young as 8–12 years of age and many, but not all, will proceed to a clinical diagnosis of dementia about four to five decades later [63]. Research suggests that sleep quality could (theoretically) scale the time in between these events. Chronic sleep restriction accelerates the build-up of beta-amyloid in mouse models of AD [64], [65]. Acting in a positive feedback loop, the resulting plaque deposition can further erode sleep consolidation in animals, yielding worse amyloid pathology and faster deterioration of cognition [65]. If this process were operational in humans, children with DS with the worst sleep problems would be expected to be at greater risk of premature cognitive decline than those maintaining good sleep health. Longitudinal efforts await to better establish the link between sleep and AD progression in the aging TD community and in aging communities with DS, and to establish whether sleep can provide a probabilistic biomarker of impending mild cognitive impairment or dementia.

The results of the present study suggest that any sleep-cognition correlations measured in children with DS likely arise without confound of improper circadian timekeeping under light and socially entrained conditions. The preservation of rest-activity rhythms in the pediatric population with DS is unique, relative to populations with other neurodevelopmental backgrounds such as Smith-Magenis syndrome (SMS) or autism. Circadian disturbance is one hallmark feature of SMS, a condition that emerges after microdeletion of the small arm of Hsa17 and haploinsufficiency for the *RAI1* (retinoic acid induced one) gene [66]. Most children with SMS exhibit inverted rhythms of melatonin secretion, sleep phase alterations, and shorter, broken sleep cycles; the phenotypes have been linked to the role of *RAI1* in regulating the molecular components of the brain's circadian clock [66], [67]. Many individuals with autism also show sleep-circadian disturbances [68]. Some studies have reported a lack of circadian variation of cortisol and melatonin secretion in cohorts of people with autism, while others have highlighted possible phase advances of early morning behavioral activity [68]. These phenotypes, too, might be linked to alterations in the molecular clock gene machinery [69]. The amalgam of data from individuals with SMS or autism suggests that circadian problems in these conditions arise from genetic interactions that prevent the molecular components of the circadian clock from oscillating as they do in TD individuals. The situation in DS is, therefore, striking: despite the overexpression of >170 genes on Hsa21, individuals with DS continue to demonstrate typical patterns of daily activity and, possibly, a properly functioning internal clock.

Altogether, the present results place a spotlight on early childhood as an important critical period for the initiation of interventions to treat sleep disorders in those born with trisomy-21 and on the necessity of adapting and popularizing long-term treatment options so that they are more engrained within the everyday care of infants and toddlers with DS. Ensuring quality sleep through the first three years of life might be a tangible treatment option for the majority of families caring for children with DS. Such interventions have the potential to optimize responses to education and cognitive outcomes.

## Financial support

Prof. Fernandez thanks Science Foundation Arizona (SFAz) and the BIO5 Institute at the University of Arizona for their generous support. The study was supported by the LuMind Research Down Syndrome Foundation and the Bill and Melinda Gates Foundation (to JOE). The UA Minority Access to Research Careers Program supported Bianca Demara (NIH MARC USTAR 5-T34-GM-008718). Additional funding from the State of Arizona and Arizona Department of Health Services was provided to Fabian Fernandez, Casandra Nyhuis, and Jamie Edgin.

## Figures and Tables

**Table 1:** Descriptive statistics for sleep-circadian measures used in the study.

	Group	
	TD	DS
*Measures of circadian robustness*		
LSP 24-h amplitude	865.54 (326.54)	873.08 (318.98)
FFT 24-h amplitude	0.207 (0.066)	0.215 (0.063)
NPCRA-IV	0.725 (0.165)	0.729 (0.190)
NPCRA-IS	0.435 (0.062)	0.455 (0.076)
NPCRA-RA	**0.935** (0.032)	0.888 (0.048)
NPCRA-M10	563.27 (153.33)	523.80 (148.53)
NPCRA-L5	17.34 (7.17)	**29.32** (11.35)
*Circadian phase markers*		
Onset	6.91 (0.96)	6.96 (**1.30**)
Onset deviation	0.64 (0.24)	0.61 (0.27)
Offset	20.86 (0.96)	20.87 (**1.36**)
Offset deviation	0.76 (0.41)	0.74 (0.44)
Acrophase	13.93 (0.91)	13.87 (**1.42**)
Acrophase deviation	0.96 (0.37)	0.89 (0.45)
*Sleep markers*		
Average sleep efficiency (%)	**82.90** (5.63)	75.86 (5.49)
Average sleep duration (minutes)	**489.66** (55.06)	453.07 (47.93)

Note: For ease of comparison, the raw group averages are shown along with standard deviations in parenthesis. Bolded numbers indicate measures that were statistically different between the groups. Please see Supplemental information for the log transformed numbers and statistical group comparisons using *t*-tests for equality of means (two-Tailed, equal and unequal variances assumed).

DS, Down syndrome; TD, typically developing; LSP, Lomb-Scargle periodogram; FFT, fast Fourier transform; NPCRA, Non-parametric circadian rhythm analysis; IV, intradaily variability; IS, interdaily stability; RA, relative amplitude; M10, Most active 10-h period of the day; L5, Least active 5-h period of the day.

**Table 2:** Summary of regression models for markers of circadian rhythms and sleep efficiency.

Dependent variable	Predictor	*B* (*SE*)	*β*	Sig.	*R*^*2*^*Δ*
LSP 24-h	Intercept	6.697 (0.046)			
	Group	0.009 (0.059)	0.01	0.874	0.00
	Age	0.012 (0.002)	0.57	**<0.001**	0.33
FFT 24-h	Intercept	−1.622 (0.038)			
	Group	0.038 (0.048)	0.06	0.429	0.01
	Age	0.010 (0.001)	0.59	**<0.001**	0.34
Intradaily variability	Intercept	−0.357 (0.027)			
	Group	0.013 (0.034)	0.03	0.696	0.00
	Age	−0.010 (0.001)	−0.72	**<0.001**	0.51
Interdaily stability	Intercept	0.436 (0.009)			
	Group	0.018 (0.012)	0.13	0.134	0.02
	Age	0.002 (0.000)	0.49	**<0.001**	0.24
NPCRA relative amplitude	Intercept	−0.067 (0.006)			
	Group	−0.054 (0.008)	−0.49	**<0.001**	0.23
	Age	0.001 (0.000)	0.47	**<0.001**	0.22
NPCRA M10 activity	Intercept	566.001 (18.215)			
	Group	−43.974 (23.411)	−0.14	0.063	0.01
	Age	5.387 (0.675)	0.61	**<0.001**	0.37
NPCRA L5 activity	Intercept	2.769 (0.061)			
	Group	0.534 (0.079)	0.54	**<0.001**	0.29
	Age	−0.005 (0.002)	−0.19	**0.019**	0.04
Sleep efficiency	Intercept	82.944 (0.822)			
	Group	−7.102 (1.057)	−0.54	**<0.001**	0.28
	Age	0.082 (0.03)	0.22	**0.008**	0.05
Phase angle onset	Intercept	1.924 (0.025)			
	Group	0.000 (0.033)	0.00	0.992	0.00
	Age	0.001 (0.001)	0.08	0.412	0.05
Phase angle offset	Intercept	3.037 (0.009)			
	Group	−0.001 (0.011)	−0.01	0.900	0.00
	Age	0.001 (0.000)	0.22	**0.020**	0.05
Phase angle acrophase	Intercept	2.632 (0.013)			
	Group	−0.007 (0.017)	−0.04	0.671	0.00
	Age	0.000 (0.000)	0.00	0.973	0.00
Onset variation	Intercept	−0.524 (0.063)			
	Group	−0.058 (0.081)	−0.07	0.471	0.01
	Age	−0.005 (0.002)	−0.22	**0.024**	0.05
Offset variation	Intercept	−0.405 (0.09)			
	Group	−0.065 (0.115)	−0.05	0.574	0.00
	Age	0.002 (0.003)	0.05	0.573	0.00
Acrophase variation	Intercept	−0.109 (0.07)			
	Group	−0.127 (0.09)	−0.14	0.159	0.02
	Age	0.000 (00.003)	0.00	0.963	0.00

Note: Age is represented in months. The intercept reflects the estimated value for a typically developing child at 30 months, the mean age of the sample. Bolded numbers indicate measures that were statistically significant for group or age effects. Note that several of the variables were transformed using a natural log prior to analyses. LSP, Lomb-Scargle periodogram; FFT, fast Fourier transform; NPCRA, Non-parametric circadian rhythm analysis; M10, Most active 10-h period of the day; L5, Least active 5-h period of the day.

## Appendix A Supplementary data

Table 1. Tests of Normality.

Table 2. Group Statistics.

Table 3. *T*-tests.

Table 4. Regression Model Summaries.
